# Using a Novel Gameplay Intervention to Target Intrusive Memories After Work-Related Trauma: Iterative Qualitative Analysis of Intensive Care Unit Staff Experiences

**DOI:** 10.2196/47458

**Published:** 2024-02-29

**Authors:** Priya Patel, Susan Brown, Boliang Guo, Emily A Holmes, Lalitha Iyadurai, Jonathan Kingslake, Julie Highfield, Richard Morriss

**Affiliations:** 1 NIHR ARC East Midlands University of Nottingham Nottingham United Kingdom; 2 NIHR MindTech MedTech Co-operative University of Nottingham Nottingham United Kingdom; 3 Institute of Mental Health University of Nottingham Nottingham United Kingdom; 4 Department of Women’s and Children’s Health Uppsala University Uppsala Sweden; 5 P1Vital Products Ltd Wallingford Oxfordshire United Kingdom; 6 Intensive Care Society London United Kingdom; 7 NIHR Nottingham Biomedical Research Centre Nottingham United Kingdom

**Keywords:** intensive care, posttraumatic stress disorder, PTSD, qualitative research, intervention study, health care professionals, digital intervention, staff well-being, pandemic, intrusive memories, work-related trauma, mobile phone

## Abstract

**Background:**

Many intensive care unit (ICU) staff experience intrusive memories following work-related traumatic events, which can lead to long-term mental health outcomes and impact work functioning. There is a need for interventions that target intrusive memories in this population; however, factors such as mental health stigma and difficulty in fitting interventions into busy schedules can pose barriers. The Brief Gameplay Intervention For National Health Service Intensive Care Unit Staff Affected By COVID-19 Trauma (GAINS) study tested a brief, digital imagery-competing task intervention (including computer gameplay) with the aim of reducing the recurrence of intrusive memories, which holds promise for overcoming some of these barriers.

**Objective:**

This substudy aims to explore barriers and facilitators to the uptake and practical use of the intervention by ICU staff, along with its acceptability, and iteratively explore the impact of intervention optimizations to further refine the intervention.

**Methods:**

The GAINS study is a randomized controlled trial comparing access to a brief digital imagery-competing task intervention for 4 weeks with usual care followed by delayed access to the intervention. The participants were ICU staff who worked during the COVID-19 pandemic and experienced intrusive memories. All participants were sent a questionnaire at 4 weeks to gather data about intervention acceptability. Nested within the randomized controlled trial, a subset of 16 participants was interviewed, and data were analyzed using thematic analysis drawing from a framework approach.

**Results:**

Both quantitative and qualitative data indicated high acceptability of the intervention. Intervention use data show that, on average, staff were able to target approximately 73% (3.64/4.88) of their intrusive memories and engaged with the Tetris component for the full 20 minutes per session. Overall, on the acceptability questionnaire, staff found the intervention easy to use, helpful, and highly acceptable. The interviews generated four themes: approach to the intervention, positives of the intervention, negatives of the intervention, and improvements and optimizations. Findings highlighted barriers that ICU staff experienced: stigma, feeling weak for seeking help, not wanting colleagues to know they were struggling, and skepticism. However, they provided suggestions on how barriers could be overcome and discussed the advantages of the intervention when compared with other treatments. Although participants described many positive aspects of the intervention, such as being easy to use, enjoyable, and leading to a reduction in the frequency or intensity of intrusive memories, they also raised practical issues for implementation.

**Conclusions:**

The intervention has the potential to overcome stigma and reduce the frequency of intrusive memories after traumatic events among ICU staff. Further refinement is needed to improve the adoption and reach of this intervention. A limitation is that we could not interview the National Health Service staff who were unable or unwilling to take part in the trial.

## Introduction

### Background

Following exposure to a psychologically traumatic event (eg, witnessing a severe injury or death) [1], intrusive memories are common, particularly in the first few days and weeks. Intrusive memories are emotional, intrusive, and primarily visual memories of the traumatic event that pop unbidden into the mind [1], that is, it takes the form of mental imagery. When they intrude repeatedly into mind, they comprise a “core clinical feature” of posttraumatic stress disorder (PTSD) [1]. Frontline health care staff are particularly at risk, with 65% of emergency nurses reporting having intrusive memories of work-related traumatic events prepandemic, such as the death of a patient [2]. For some individuals, intrusive memories persist for more than a month and thus become a core symptom of PTSD [3]. It has been known for some time that frontline health care staff experience repeated exposure to potentially traumatic events [4-7], even before the pandemic. This exposure was even worse during the COVID-19 pandemic, with 5 times more UK health care staff reporting PTSD symptoms, such as bothersome intrusive memories, in 2020 than in 2015 [8,9]. In this study, we will focus on intensive care unit (ICU) staff working during the pandemic, although it is assumed that this has wider relevance as trauma exposure and intrusive memories also affect other staff groups, and experiencing trauma was also prevalent prepandemic. A key difference with the pandemic was the increased frequency of trauma exposure for this group.

PTSD symptoms, such as intrusive memories, are associated with poorer long-term physical and mental health outcomes [10]. There is a great cost for patients and society when frontline staff are affected, with 27% of health care staff who reported PTSD symptoms believing that their work functioning was negatively impacted [6]. Furthermore, there are problems with staff shortages and dropouts, with PTSD symptoms causing 20% of staff to consider a job change [6]. Mental health problems remain the leading cause of sickness absence in the National Health Service (NHS) [11]. Owing to these factors, the mental health of frontline health care staff exposed to traumatic events is a major priority internationally [12].

### Prior Work

A novel approach in this area is the development of a brief mechanism-driven behavioral intervention to reduce intrusive memories [13-15]. This brief imagery-competing task intervention for established intrusive memories after trauma consists of a reminder cue to the traumatic event, followed by playing the computer game Tetris for 20 minutes with instructions to use mental rotation during gameplay [16]. The principles of the intervention are informed by the neuroscience of memory storage and updating (so called consolidation and reconsolidation) [17,18] and cognitive task interference [19]. The hypothesis is that the memory consolidation or reconsolidation process of a traumatic event can be disrupted by engaging in visuospatial demanding tasks, for example, Tetris, and reduce the frequency of the intrusive memories. Randomized controlled trials (RCTs) have shown that this type of intervention approach can reduce the frequency of intrusive memories soon after trauma exposure in women after traumatic childbirth [20] and after a traumatic motor vehicle accident [21,22]. In addition, the intervention has recently been found to reduce established and older intrusive memories in case series studies with patients with chronic PTSD [23], refugees [24], and most recently NHS staff exposed to work-related trauma [16,25]. In particular, ICU staff (those working in intensive care, intensive therapy, and high-dependency units) face repeated exposure to trauma as an inevitable and intrinsic part of the work setting [2]. Now that the adverse effects on staff health and well-being are becoming better recognized [12], it is imperative to find ways to address these needs.

This study is part of a Brief Gameplay Intervention For National Health Service Intensive Care Unit Staff Affected By COVID-19 Trauma (GAINS) study (ClinicalTrials.gov: NCT04992390) for health care staff in the United Kingdom who faced trauma exposure as part of their work during the COVID-19 pandemic. The intervention used a brief imagery-competing task intervention with the aim of reducing and preventing the recurrence of intrusive memories from work-related trauma exposure.

There are many barriers to the implementation of both digital and face-to-face mental well-being interventions in health care staff, specifically owing to the complexity of the role and organization of health care [26]. Personal barriers to uptake by health care staff include a perceived lack of ownership when they feel an intervention is not driven by them; feeling as though they are obliged to participate; and practical barriers to participation, such as cost, time commitments, and age [26,27]. In setting up the GAINS study in collaboration with the Intensive Care Society (ICS) in the United Kingdom, it became clear that time commitment is of particular importance, as the nature of their role as health care staff means they are working in busy and pressurized environments, even more so given staff shortage problems. Staff have indicated that financial barriers have created a perception that spending priorities prioritize patients’ needs over the well-being of the staff [26,27]. The situation is made more complex by organizational barriers, including changes in senior management, managers being perceived as inaccessible, ongoing organizational changes and restructuring, and the influence of target-driven cultures [26]. A lack of suitable infrastructure to support digital health interventions has also been found to be a barrier to use [28], such as a lack of available computers or internet connection, which could be a potential barrier for staff working in hospital settings.

There are also huge barriers owing to stigma and inclusivity, with many health care professionals experiencing shame for struggling with their mental health [29]. A literature review found that, within the nursing population specifically, many nurses felt that they needed to keep their mental health a secret owing to fear of being judged by their colleagues [30]. This was not only because of fear of what others may think but also because of self-directed stigma, with 21% of nurses struggling with their mental health believing that this was because of a personality weakness or character defect [30]. Therefore, digital health interventions may pose a key strength because they can be accessed independently and from any location, thereby providing users with privacy and anonymity [28]. This is in contrast to, for example, attending mental health services for psychological therapy or medication. In addition, it is vital to ensure that study samples are representative, as intersectionality plays a role in this, with health care professionals from ethnic minority backgrounds experiencing increased workplace discrimination and harassment [31]. Barriers and facilitators to the implementation of mental health interventions for NHS staff will occur at the organizational and individual levels. Therefore, it is necessary to consider the barriers and facilitators that may exist at multiple levels.

### This Intervention

The GAINS intervention used a secure web-based mental health and well-being platform (i-spero, P1vital Products Ltd) to allow participants to access the intervention on a smartphone, tablet, or computer. Participants had an initial guided session with a researcher over Microsoft Teams, in which they were provided with step-by-step instructions on how to use the intervention as well as explanatory videos and multiple-choice questions. Participants were assisted in briefly listing their intrusive memories during the initial session (hotspots). Subsequently, they were prompted to recall the image associated with 1 specific intrusive memory. They played the Tetris game for 20 minutes using mental rotation, in which they had to imagine how to rotate the next Tetris piece so that it could fit in the existing structure. The intervention took a total of approximately 25 minutes each time, and they could target different memories on different days. Finally, they were trained in using the i-spero (P1vital Products Ltd) platform to monitor intrusive memories in daily life [25].

The underlying principle of the intervention is its imagery-based nature and that it can be used regardless of the content of the intrusive memory (a motor vehicle accident or witnessing a patient’s death when working in the ICU). The intervention is used once per different intrusive memory, so it can also be used by someone who has experienced a single traumatic event or multiple ongoing traumatic events. It can also be used for new intrusive memories that develop during the trial. Owing to the nature of their roles, participants work in an environment where trauma can be a frequent occurrence. Therefore, choosing an intervention that can address the specific challenge of recurring and frequent trauma is crucial.

This intervention holds particular promise for overcoming some of the mentioned barriers to the implementation of mental well-being interventions after trauma in ICU settings [25]. For example, rather than focusing on a mental health diagnosis such as PTSD, the entry to accessing the intervention is a simpler index problem, namely, having intrusive memories of the traumatic event. It is brief (1 guided intervention session of 1 hour, followed by self-guided use of approximately 25 minutes per session, whereby the aim is 1 session per different intrusive memory). It is digital and can be used flexibly in different locations (eg, on a smartphone during a commute) and may have lower stigma than attending mental health services (as the intervention involves a digital task including a computer game rather than, for example, talking to a trained therapist). As the intervention can be used for new intrusive memories as they arise, it is well suited for health care staff facing repeated or ongoing trauma in their jobs. Finally, as the intervention requires minimal therapist resources, it has the potential to be more cost-effective and scalable than current evidence-based interventions that require more contact.

### Aims

This qualitative substudy as part of the GAINS study had the following aims:

- To explore barriers and facilitators to the uptake and use of the imagery-based competing task intervention to reduce intrusive memories of work-related trauma in ICU staff, along with its acceptability.
- To iteratively explore the impact of optimizations made to the intervention to address some of the barriers (and enhance facilitators), allowing us to then further refine the intervention for future use by ICU staff.

## Methods

### Design

The GAINS study is an RCT comparing immediate access to a brief digital imagery-competing task intervention for 4 weeks (the immediate intervention arm) versus receiving usual care for 4 weeks, followed by delayed access to the intervention for an additional 4 weeks (the delayed intervention arm). This manuscript contains quantitative descriptive data from an acceptability survey completed 4 weeks after the first intervention session as well as data from the intervention itself on uptake and completion of the intervention. This descriptive data are provided as contextual information for the qualitative findings, which explore in more detail barriers and facilitators to the uptake, completion, and overall acceptability of the study. The qualitative analysis draws on 2 sources of data collection: interviews from a maximum variance sampling method in a subset of participants and free-text feedback that was sought from all participants completing the acceptability survey. The narrative feedback was used to triangulate the qualitative interview data and to check whether there were additional themes or subthemes in addition to those emerging from the qualitative interview analysis. As this paper details the findings of a qualitative substudy nested within an RCT of an intervention, details of the RCT method, intervention, and acceptability survey are provided first, followed by the qualitative substudy method.

### Ethical Considerations

GAINS study part 1 received a favorable opinion from Wales Research Ethics Committee (REC) 6 on May 21, 2021 (REC Reference 21/WA/0173 and Integrated Research Application System project ID number 297063). There were 4 non-substantial amendments made to the Interview Topic Guide - non-substantial amendment 1 on July 21, 2021; non-substantial amendment 3 on Oct 5, 2021; non-substantial amendment 5 on Nov 12, 2021; non-substantial amendment 9 on Jun 8, 2022. The purpose of these amendments was to slightly amend wording of questions, gather information about which NHS Trust the participant worked in and gather thoughts on the optimised version of the intervention.

### Recruitment for the GAINS Study RCT

The participants were ICU staff who worked during the COVID-19 pandemic and experienced intrusive memories as a result. Participants were recruited through ICS membership and existing social media followers supplemented by targeted advertisements on social media (eg, Facebook and Twitter) [16,25]. The advertisement email contained a link to the study website, where interested individuals were able to read a study summary, including the participant information sheet (Multimedia Appendix 1), and watch a video explaining intrusive memories in further detail. The study website also included a link to the 10-item prescreening eligibility questionnaire.

### Inclusion and Exclusion Criteria of the GAINS Study RCT

Potential participants met the inclusion criteria if they were aged ≥18 years; able to read, write, and speak in English; worked in a clinical role in an NHS ICU or equivalent during the COVID-19 pandemic; experienced at least 1 traumatic event related to their work during the COVID-19 pandemic; meeting criterion A of the *Diagnostic and Statistical Manual of Mental Disorders, Fifth Edition* criteria for PTSD: “exposure to actual or threatened death, serious injury, or sexual violence” by “directly experiencing the traumatic event(s)” or “witnessing, in person, the event(s) as it occurred to others”; experience intrusive memories of the traumatic event or events; experienced at least 3 intrusive memories in the week before screening; have internet access; were willing and able to provide informed consent and complete study procedures (including briefly listing their intrusive memories [without going into any detail] and playing the computer game Tetris with particular mental rotation instructions and completing a web-based intrusive memory diary); and were willing and able to be contacted by the research team during the study period. Potential participants were excluded if they had <3 intrusive memories during the run-in week.

Individuals who met the inclusion criteria were given the participant information sheet again, along with the opportunity to ask questions to the investigator, their general practitioner, or other independent parties to make an informed decision about whether to participate. If they were still interested in participating in the study, a researcher arranged a time to contact them by phone or video call to obtain informed consent. The participant and researcher completed, signed, and dated the consent form using a simple electronic signature via email, which included providing permission to be contacted for the qualitative interview component of the study. The consent form was retained electronically in a secure format, and participants were emailed a copy for their records.

### Study Procedures of the GAINS Study RCT

After providing informed consent, participants were asked to complete a daily web-based intrusive memory diary for a run-in period of 1 week. Each day, the participants were asked to indicate if they had any intrusive memories and, if so, how many. Participants who met the eligibility criterion of having ≥3 intrusive memories in the run-in week were randomly allocated to either the immediate intervention arm or the delayed intervention arm in a 1:1 overall ratio. Participants were randomized through the P1vital electronic Participant Reported Outcome system (P1vital Products Ltd), and the outcome assessment was completed remotely by the participants (independently of the research team) on this platform. The qualitative team was independent of randomization, delivery of the intervention, and assessment of the quantitative outcomes. The immediate intervention group received the intervention immediately for 4 weeks, whereas the delayed group received usual care for 4 weeks, followed by the 4-week-long intervention.

### Intervention Being Trialed

The brief digital intervention was delivered through a secure web platform that participants accessed on their smartphone or other internet-enabled device (refer to the studies by Iyadurai et al [25] and Ramineni et al [16] for complete details). Participants were provided with an initial session guided by a clinical psychologist or delegated researcher to run through the intervention as well as follow-ups and support available throughout the intervention. During the initial session (approximately 1 hour), participants were asked to briefly list the different intrusive memories they have and choose the one they wish to target first. They were then asked to complete the intervention, which included several key components: (1) the participant was asked to briefly bring to mind the intrusive image as a reminder to the specific memory, (2) they received instructions on how to play the computer game Tetris using “mental rotation,” and (3) they were asked to play Tetris using mental rotation for at least 20 minutes. During the intervention, participants were asked to rate how distressed they are feeling on 3 occasions, to rate the vividness of the image that is brought to mind, and to rate how much they were able to follow mental rotation instructions, to assess adherence to the instructions. After this first session, participants were able to use the intervention as many times as they liked over the next 4 weeks (eg, to target any other intrusive memories on their list or those that recur—approximately 20 min/session). The system logged intervention use data, including the number of intrusive memories on the participant’s memory list, number of intrusive memories targeted with the intervention, number of times the intervention was started and used by the participant, and the total time spent playing Tetris.

### Data Collection

#### Intervention Feedback Questionnaire Procedures and Analysis

All participants were sent the Intervention Feedback Questionnaire (IFQ; Multimedia Appendix 2) 4 weeks after the first intervention session to gather information about intervention acceptability. The quantitative data from this questionnaire were analyzed descriptively by an independent and blinded statistician (BG), and the uptake and use data were analyzed by the P1Vital data management team. The qualitative research team only received the questionnaire free-text data after completing the qualitative interview analysis. PP categorized any free-text responses that fitted our qualitative themes and subthemes. SB and RM then reviewed these categorizations, and the very few instances of discrepancies were discussed to create the final structure. These data were analyzed descriptively in terms of frequency of response as a proportion of all participants and also of those who made any response at all. We then examined any data that did not fit any of our themes and considered whether there was enough detail to identify the data as a new theme or subtheme within the overall analysis. If a comment was too vague or general to determine whether it fitted the current thematic or subthematic structure or not, we did not include it in the analysis. As there is a separate trial outcome manuscript that explores the effectiveness of the intervention on a variety of outcomes [16,25], we excluded comments related specifically to the outcomes measured in the trial protocol.

#### Nested Qualitative Study

##### Recruitment

Upon completing the 4-week intervention, participants who had previously consented to be contacted for the interview component of the study were asked if they would like to take part in a qualitative interview with a researcher via an audio or a video call. Details of interested participants were stored on a password-protected file, and a researcher used selective sampling to contact participants from diverse backgrounds (age, gender, ethnicity, job role, and location) to schedule an interview.

##### Interview Schedule

This semistructured interview consisted of several questions designed to gain an in-depth understanding of participants’ experience of using the intervention, including acceptability, improvement suggestions, training or psychoeducation materials, potential barriers or facilitators to recruitment and uptake, and support needed for remote intervention delivery (Multimedia Appendix 3).

##### Procedures

Before commencing the interview, the researcher confirmed consent to audio record the interviews using a digital voice recorder, and the participants were reminded of the option to withdraw at any point. The interviews lasted approximately 30 minutes, and the audio recordings were immediately transferred to a password-protected laptop and deleted from the voice recorder. The password-protected files were then sent for transcription and anonymized.

##### Data Analysis

The interview data were analyzed using thematic analysis [32] and drawing from the framework approach by Ritchie et al [33] and Spencer et al [34]. A hybrid approach was used, where themes were generated inductively (from the data) and deductively (based on core areas of interest). This analysis approach was used as the study was exploratory at this stage, so it was important to understand the feasibility and acceptability of the intervention alongside core experiential elements that were not anticipated.

Steps of the analysis included the following:

- Familiarization with data (noting arising concepts and patterns)
- Generating an initial coding framework (iteratively and through team discussion)
- Coding of all transcripts
- Reviewing the content of codes in depth; identifying themes and subthemes; and exploring coherence, variation, consistency, and prevalence
- Creation of mind maps, showing how themes and subthemes fit together and interact, and identifying linkages

In relation to the coding processes, 1 researcher (PP) received interview transcripts, anonymized these transcripts, entered them into NVivo12 (Lumivero), and coded them. A second researcher (SB) coded a sample of transcripts, followed by discussions regarding any discrepancies. Once coding was completed, codes were explored in-depth by PP, who created summaries of coded content to allow the exploration of themes and subthemes. SB repeated the same process on a sample of codes to check for consistency and any discrepancies. PP and SB then worked together to develop and prioritize themes and categorize subthemes according to prevalence.

The analysis initially focused on the barriers and facilitators to using the intervention and how helpful the intervention was for participants, before going on to look at how it could be optimized for future participants and circulated wider.

## Results

### Overview

In total, 86 participants took part in the GAINS study RCT, 43 (50%) of whom were randomized to the delayed arm and 43 (50%) to the immediate arm [16,25]. Of the 73 participants approached to participate in an interview, 61 (83%) consented to being contacted, 1 (1%) declined, and 11 (15%) did not respond. The mean number of different intrusive memories listed by each participant was 4.88 (SD 2.17), and the mean number of different intrusive memories targeted per participant was 3.64 (SD 2.04). Further intervention use data are presented in Table 1. This shows that participants used the intervention an average of 7 times over the 4-week period and spent approximately 20 minutes and 54 seconds playing Tetris per session. They were able to target an average of 73% (3.64 targeted/4.88 total) of the intrusive memories on their list using the intervention.

**Table 1:** Data on intervention use.

	Values, median (IQR; range)
Proportion of intrusive memories targeted from list (%)	73 (50-100; 8.33-100)
Number of times intervention used	7 (5-12.75; 1-44)
Total time spent playing Tetris per use	20 min 54 s (20 min 22 s-22 min 8 s; 11 min 48 s-31 min 20 s)

The median number of intrusive memories dropped from 14.50 (IQR 10.0-21.50) preintervention to 1.00 (IQR 0.0-3.0) postintervention in the immediate arm group. The median number of intrusive memories dropped from 10.00 (IQR 6.0-17.0) preintervention to 1.00 (IQR 0.0-2.50) postintervention in the delayed arm group. Furthermore, following intervention use, there was a significant reduction in PTSD symptoms (*P*<.001), insomnia (*P*<.001), and anxiety (*P*=.02) and an increase in work functioning (*P*<.001) and well-being (*P*<.001) [16,25].

Of the 86 participants, 84 (98%) completed the IFQ, which contained a mixture of quantitative (scale) and qualitative (free text) response options. The quantitative data will be provided initially, and the qualitative data will be discussed alongside the interview findings. The IFQ respondent demographics are shown in Table 2. Participants’ responses to each quantitative item of the IFQ are presented in Table 3.

**Table 2:** Demographics of the Intervention Feedback Questionnaire respondents (N=84).

Demographic factors			Respondents, n (%)
**Gender**			
	Female	69 (82)	
	Male	15 (18)	
	Other	0 (0)	
**Highest educational qualification**			
	Bachelor’s degree or equivalent	47 (56)	
	Master’s degree	24 (29)	
	Doctoral degree	6 (7)	
	Sixth form or equivalent (to age 18 y)	4 (5)	
	Prefer not to answer	2 (2)	
	Secondary school (up to age 16 y)	1 (1)	
**Marital status**			
	Married or cohabiting	53 (63)	
	Single	26 (31)	
	Divorced or separated	4 (5)	
	Living apart from partner	1 (1)	
**Ethnicity**			
	Asian (Indian)	5 (6)	
	Asian (any other Asian background)	1 (1)	
	Black (African)	2 (2)	
	White (British)	36 (43)	
	White (Irish)	2 (2)	
	White (any other White background)	23 (27)	
	Mixed (any other mixed background)	2 (2)	
	Other (any other ethnic group)	1 (1)	
	Unknown (not stated)	12 (14)	
**Occupational status**			
	Working full time	66 (79)	
	Working part-time	16 (19)	
	Other	1 (1)	
	Sick leave	1 (1)	

**Table 3:** Mean score for each Intervention Feedback Questionnaire (IFQ) item.

IFQ item	Score, mean (SD)
IFQ0101: How easy did you find it to use the intervention? (0=not at all and 10=very)	8.59 (1.93)
IFQ0102: How helpful did you find the intervention? (0=not at all and 10=very)	8.23 (2.32)
IFQ0103: How burdensome did you find the intervention? (0=very and 10=not at all)	6.48 (2.70)
IFQ0104: How distressing did you find the intervention? (0=very and 10=not at all)	7.07 (2.63)
IFQ0105: Overall, how acceptable did you find the intervention? (0=not at all and 10=very)	8.50 (1.88)
IFQ0108: If you were having intrusive memories in the future, how willing would you be to use the intervention if it was offered to you as something that would help? (0=not at all and 10=very)	8.79 (2.11)
IFQ0109: If a colleague or friend was having intrusive memories, how confident would you be in recommending the intervention to them? (0=not at all and 10=very)	8.43 (2.22)
IFQ0110: How much do you feel that this intervention could be used within NHS^a^ Trusts or health care organizations to support staff who have experienced work-related traumatic events? (0=not at all and 10=very)	8.38 (2.24)
IFQ0113: Total score (0-80)	64.46 (12.51)

^a^NHS: National Health Service.

Table 2 with IQR results shows that the vast majority of participants who took part in the trial were female, working full time, educated to degree level, married or cohabiting, and from a White British or White non-British background. The sample included male, part-time, less educated, single or separated, and ethnic minority staff (from Asian, African, and mixed ethnicity backgrounds). After 4 weeks, most staff reported that the intervention was easy to use, helpful, burdensome, distressing only to a mild degree, and highly acceptable. In the future, they were highly willing to use the intervention again if it was offered to them, highly confident in recommending it to colleagues, and believed strongly that it would help other staff in health care settings who experience trauma.

In the nested qualitative interview study, 16 participants were interviewed. Table 4 shows the demographics of the interview sample. The mean age of the interviewees was 39.4 (SD 8.4) years. Following the first 8 interviews, maximum variance sampling was used to select interviewees from a range of backgrounds. This included a range of ages, genders (predominantly female), ethnicities, job roles (predominantly nurses and consultants), geographical locations, and number of intrusive memories at baseline (range 5-44).

**Table 4:** Demographics of the interview sample (N=16).

Demographic factors		Interview sample, n (%)
**Age** **(y)**		
	18-25	0 (0)
	26-35	3 (19)
	36-45	7 (44)
	46-55	4 (25)
	>56	1 (6)
	Not known	1 (6)
**Number of intrusive memories at baseline**		
	1-5	1 (6)
	6-10	2 (13)
	11-15	3 (19)
	16-20	4 (25)
	21-25	4 (25)
	26-30	1 (6)
	31-35	0 (0)
	36-40	0 (0)
	41-45	1 (6)
**Gender**		
	Female	11 (69)
	Male	4 (25)
	Prefer not to say	1 (6)
**Ethnicity**		
	African	2 (13)
	Filipino	1 (6)
	Indian	2 (13)
	Spanish Colombian	1 (6)
	White	8 (50)
	Mixed race	1 (6)
	Prefer not to say	1 (6)
**Job roles**		
	Nursing	9 (56)
	Consultant	3 (19)
	Pharmacist	1 (6)
	Anesthetist	1 (6)
	Physician	1 (6)
	Dietitian	1 (6)

The final 4 (25%) of the 16 interview participants who participated in the study received an optimized intervention, as this was a Bayesian adaptive RCT. The intervention was optimized as the study progressed over time [16,25] following feedback from previous participants. These optimizations included adding graphs to allow participants to see their own data for each intrusive memory, adding a video at the end of the first guided session to reinforce how to use the intervention independently, and adding an additional reminder cue in the first guided session to ensure that the memory was in their mind just before they played Tetris [16]. The topic guide (Multimedia Appendix 3) was, therefore, amended to include questions about participants’ thoughts on what was required to access the intervention independently as well as their thoughts on the added graphs and reminders.

The themes and subthemes are outlined in Textbox 1, and example quotes illustrating each theme are provided in the text below.

**Thematic structure.**

**Themes and subthemes**

- Attitudinal and emotional responses
  Experiencing mental health symptoms as a health care professional
    - Stigma and imposterhood
    - Value of anonymity
  Using a novel intervention
    - Skepticism
    - Understanding how it works is helpful
- Positives of the intervention
  Tracking and intervening to reduce intrusive memory frequency and intensity
  Intervention is easy to use and enjoyable
  Intervention is more convenient than psychological therapies
  No need to discuss intrusive memories
  No side effects of the intervention
  Cognitive and emotional coping
- Negatives of the intervention
  No opportunity to discuss intrusive memories in detail
  Unclear whether memories are spontaneous
  Technological issues
  Difficult to find time to use the intervention
- Improvements and optimizations
  Difficult to focus on mental rotation
  How to increase focus on mental rotation
  Researcher support is important
  How to access the intervention independently
  How to aid incorporation of the intervention into participant lifestyle
  Other intervention improvement suggestions

### Attitudinal and Emotional Responses

#### Experiencing Mental Health Symptoms as a Health Care Professional

##### Stigma and Imposterhood

Choosing whether to participate in the intervention was compounded by barriers, such as stigma surrounding mental health in ICU staff. Many participants described feeling weak for seeking help and not wanting colleagues to know that they were struggling:

I’m a healthcare professional and I do see these things all the time, but then in one way you think these are the things that will never affect you. You help the others, because I’ve always been caring and empathetic with them, but at the same time you don’t think you can be the patient.

001

But critical care nursing has this kind of almost elitism kind of approach. And the moment you have a little bit of a weakness that’s then seen negatively and your almost- you kind of feel that someone’s going to think that you can’t do your job.

008

sense of imposter-hood with these. Do I really- Or do I- Are my intrusive memories bad enough? Surely other people have worse ones, therefore- It was helpful getting rid of that feeling.

003

##### Value of Anonymity

###### Overview

In addition, participants stressed how emphasizing the anonymity of this intervention could help reduce the impact of mental health stigma on taking part in the study:

Yes I think highlighting it being anonymous and it doesn’t get reported back to work would probably make people more likely to use it. And the fact that they can – you know if they can access it on their personal emails and not work emails and things like that that would probably make people more likely to use it.

013

###### IFQ Data

Despite this, when asked how the intervention could be improved in the feedback questionnaire, 3 participants provided suggestions that required workplace involvement in the intervention:

Involve managers to support the staff.

IFQ004

Time out of work to do it.

IFQ049

For it to [be an] option within occupational health because it has impacted on NHS workers that went through the pandemic.

IFQ040

#### Using a Novel Intervention

##### Skepticism

###### Overview

Participants discussed their initial skepticism when approached with information about this intervention, as its novelty, simplicity, and game design caused them to doubt its effectiveness:

Well when I first heard about it I was very, very sceptical, because yes I know that games focus your mind on something else. But I just wasn’t convinced that it was going to do anything.

005

###### IFQ Data

Skepticism toward the intervention was also mentioned by a couple of participants in the feedback questionnaire when asked what they found useful about the intervention:

Amazing. I genuinely did not expect it to work.

IFQ012

The intervention definitely targeted some of those intrusive memories popping up, working much faster than I had expected it to.

IFQ009

##### Understanding How It Works Is Helpful

###### Overview

Therefore, participants described how increasing their understanding about how the intervention works during initial communication is important to reduce the initial skepticism about intervention efficacy:

[Once] you get over people being aware of what it’s trying to do. You know, it’s not, for example, it’s not just a distraction, it’s not trying to distract you from memories and thoughts, but this is- Try this because there is evidence as to how this will work.

010

People might be a little bit sceptical that it would work. Well especially with nursing… as part of your training you do modules on research so I think if people are shown the research and shown that it works they’re more likely to use it.

013

Participants suggested providing previous research and testimonials from other ICU staff during initial communication to help normalize intrusive memories and thereby encourage more professionals to seek help:

[Should] have said, look, I also have the same problems, have a personal story like that, and see somebody’s journey. Saying like, you stick with a little work and I think it reassures a little bit and I think it relates a little bit nicer to you mentally than just a- you know a scientific paper which is very objective and impartial.

011

###### IFQ Data

This was further highlighted by a couple of participants in the feedback questionnaires, when asked how the intervention could be improved as something that could be offered to health care staff to help reduce intrusive memories after a work-related traumatic event:

Provide evidence and testimonies from the research participants.

IFQ018

To make people aware of it and show success results.

IFQ006

### Positives of the Intervention

Participants discussed various positive effects of the intervention, including its effect on them, and also when compared with other treatments (which was a line of questioning).

#### Tracking and Intervening to Reduce Intrusive Memory Frequency and Intensity

##### Overview

Participants described how the intervention was helpful in reducing the frequency or intensity of intrusive memories. Moreover, they found it helpful to track their intrusive memories, as it allowed them to notice reductions and patterns and reinforced intervention use:

It was like being back inside the situation again. I could hear the voices...I was surrounded by the scenario again. And then it became just images of like trying to foreseeing those things again. And now it is just a cloud. There is nothing there.

>015

Because then you can see the trend and then you think oh, actually I haven't had any memories for three days or whatever. So, that kind of like spurs you on a bit and then you're like oh, it’s working, yes that was useful.

006

Recording the frequency of the intrusive memories and realising that they’re actually going down, it just reinforces that it is a good tool to use for that purpose.

002

##### IFQ Data

In total, 30% (25/84) of the feedback questionnaire respondents also described how the intervention helped to reduce the frequency and intensity of intrusive memories as well as how some of these memories did not return:

That it actually helped reduce the frequency of the memories.

IFQ011

The intervention definitely targeted some of those intrusive memories popping up...causing the memories to become less frequent as well as less distressing and vivid when they did appear.

IFQ009

It helped to reduce my intrusive memories. It gave me something active and engaging to do if I felt distressed by my memories.

IFQ035

#### Intervention Is Easy to Use and Enjoyable

##### Overview

Participants found the intervention easy to use, in part because of the clear instructions provided, and enjoyable:

I think it was all really straightforward. And for someone of my age, that’s- It must be easy, because I’m of the non-tech generation.

016

You know, it is a psychological intervention and it is helping you psychologically [laughs]. But it’s also, it is a game and it, there are some enjoyable aspects to it and it’s a good way to help yourself mentally.

006

##### IFQ Data

These positives were further emphasized in the feedback questionnaire responses, with a couple of respondents mentioning that the intervention was enjoyable and many more saying it was simple, easy to use, and intuitive:

It was enjoyable and very intuitive.

IFQ004

It’s such a simple tool to use to target these frustrating and upsetting thoughts.

IFQ001

It was straightforward and easy to use.

IFQ002

However, 1 participant also explained in the feedback questionnaire that their lack of experience with Tetris made it difficult to use the intervention:

Learning to play the game as I had not used before.

IFQ034

#### Intervention Is More Convenient Than Psychological Therapies

Participants also discussed the benefits of this treatment compared with existing treatment options for intrusive memories, such as psychological therapies. For example, unlike psychological treatment, this treatment is flexible as it does not require an appointment:

You can choose when to target those specific kind of memories rather than being reliant on, well I’ve got an appointment at 2 o’clock on this day. And there were days when I couldn’t- I didn’t have the mental capacity to just focus on kind of day to day let alone do the intervention. So I could pick and choose when I targeted those memories. (008)

It doesn’t take too much time out of the day and the emotional commitment involved is not exhausting, like some other forms of counselling I’ve had before. In fact, I feel better for it, rather than worn out. So, 20 minutes and then you can get on with things really.

003

It’s because it is more available whenever time you want. That’s the most important thing.

007

#### No Need to Discuss Intrusive Memories

##### Overview

Some participants also appreciated that this intervention did not require them to relive distressing memories in-depth, unlike in talking therapies:

The fact that you’re actually having a therapy session, but you don’t realise it. So, it can be less distressing as well, especially when you’ve got trauma, some people don’t really want to talk about trauma, they just want trauma to go away.

001

Because sometimes if you have counselling, you leave the room even worse than when you came in, because you are not thinking about something and then you have to go back to the situation and seeing and then you spend the whole day crying- I’ve had counselling before and after so much crying, you are so exhausted the rest of the day after all that.

015

##### IFQ Data

There were mixed views on this in the feedback questionnaire responses, with 1 respondent stating that the intervention was nondistressing and 3 respondents explaining that it was difficult to recall the memories as part of the intervention:

Pretty well non-distressing.

IFQ021

Didn’t want to bring some of the more difficult intrusive memories to mind.

IFQ044

Re living those memories was really difficult sometimes.

IFQ028

The only difficult thing was facing the intrusive thoughts.

IFQ025

#### No Side Effects of the Intervention

When compared with medication, the key advantages discussed were the lack of side effects, for example, on sleep or weight, and being able to directly target intrusive memories:

If you’ve got a problem, you still have the problem. Maybe the medication helps you to stay more relaxed, but it doesn’t really have an impact on intrusive memories, and things like that.

001

I have my own reservations when it comes to medication...I don’t want to take much of a time, you know in terms of (the drug affecting) sleep or waking or weight loss, things like that. Compared to medication, I was happy to undergo an intervention like this.

009

In addition to discussing some of the positive effects of the intervention compared with other treatments, 1 participant also mentioned simply appreciating the availability of an alternative option:

Personally because I’ve had talk therapy and medication and things so, I was looking for something else to try.

006

#### Cognitive and Emotional Coping

##### Overview

The participants described the positive effects of the intervention on their cognition, such as improved concentration and rational thinking at work. They also experienced positive changes in their emotions and other aspects of life, including feeling happier, more in control of their emotions, reduced anxiety, and sleep improvements:

That allowed your brain to then focus more on the important things. And my rational thinking at work seemed to improve as a consequence.

008

My attention and focusing time came back. Usually, you need focal attention. I can pay attention for extended hours, but I was not able to do that.

009

My productivity increased to the extent that people started noticing it and I became happier. I think I became my old self.

009

I was so tearful but now, if I’m tired, I feel more comfortable, more happy, more emotionally controlled.

007

Obviously, the sleep improvements come with not having the intrusive memory and being troubled by them.

002

##### IFQ Data

The feedback questionnaires offered insights into additional positive effects of the intervention, including how it enhanced focus, served as a helpful distraction, aided in relaxation, and allowed individuals to take time out of their day.

Facilitated focus and clarification of thoughts. Organised my mind.

IFQ015

The game was a great distraction.

IFQ022

Intervention itself was relaxing, forcing 20 minutes of exclusive concentration on a task providing a break from a busy day.

IFQ032

### Negatives of the Intervention

Participants mentioned some negative effects related to the use of the intervention, some of which were grounded in comparison with other treatments (which was a line of questioning) and some of which were given spontaneously. There is heterogeneity of experience, and these were not discussed by everyone, and these negative effects were grouped as follows.

#### No Opportunity to Discuss Intrusive Memories in Detail

##### Overview

Participants discussed some negative effects of this intervention compared with existing treatments. For example, some participants preferred to talk to a clinician about their intrusive memories, which this intervention did not allow:

[If] you are really troubled by one of the memories, I can imagine having direct psychological support to kind of work your way through that thought process, it is just a thought, calming you down, there’s none of that.

002

##### IFQ Data

One participant also highlighted in the feedback questionnaire that the inability to discuss intrusive memories in detail meant that the intervention should be accompanied by another support system:

Great as a distraction technique but needs to be accompanied by another support mechanism, being given a safe space to be able to talk through experiences etc.

IFQ017

#### Unclear Whether Memories Are Spontaneous

##### Overview

In addition, participants expressed difficulty in knowing whether memories of the traumatic event were actually spontaneous or simply because of being part of the study:

I think it’s really hard to try and differentiate between, am I putting this thought in my head? Because I know I am doing the GAINS Study, or- So I think like, after the first couple of days, I just had to just try and, like, ignore the research side of it. (004)

##### IFQ Data

Feedback questionnaire respondents concurred with this; with respondents explaining how processes inherent to the study, such as repeat contacts and reminder texts, could in fact remind them more about the distressing incidents:

Repeat contacts became a bit burdensome.

IFQ003

The phone calls, texts were far more frequent than I anticipated. The reminder to do the intervention at 7 am and at night reminded me of the distressing incidents and made me more distressed.

IFQ019

#### Technological Issues

##### Overview

Once participants had overcome the barriers to participating in the study (described previously), there were some environmental factors that affected their experience with the intervention. As this is a digital intervention, technological difficulties such as a lack of technological knowledge, reduced access to a device, and device differences were mentioned as issues:

The one thing I did find though, which I didn’t know, is when I occasionally had, I usually used my laptop if I was able, and you know, it was much easier to rotate the blocks. But I found on my mobile phone it was, it was very sensitive.

005

Some of us are lucky to have laptops, but like, I remember at the initial start, we were told to make sure you have your laptop, because it might not work, like, on your phone or your iPad.

012

There might be some groups that have potentially worked in different areas in part of the pandemic and they’ll be left with some of these intrusive memories that wouldn’t be so technologically fluent.

002

##### IFQ Data

This was echoed by the feedback questionnaire responses, with 1 participant explaining how there were problems owing to the multiple platforms and the inability to access the intervention offline. However, there were mixed views on how well the intervention worked on mobile phones:

Multiple platforms, not an intuitive website...and it would time out if you were on a train/went offline.

IFQ002

Didn’t work too well on my phone.

IFQ016

I would not improve the intervention itself as it is very accessible and able to play on phone and computer.

IFQ037

#### Difficult to Find Time to Use the Intervention

##### Overview

In addition to technological difficulties, participants described how it could be difficult to find time to use the intervention because of their busy schedules, shift patterns, and other responsibilities outside of work:

[Found] it a bit difficult to find the time with this- I’ve got a small child, I’m working almost close to full-time. My husband is a [profession name], so he does a lot of out of hours work, so a lot of the childcare and toing and froing, that comes to me.

002

[Occasionally] I had to do it just before I went to bed after a busy day. And I didn’t like doing that because I try and reduce my mobile phone usage or computer usage late at night.

005

Particularly about doing it during the day at work, made you think, well that’s fine, I’ve managed to push things, cleared everything up, I’ll be fine for half an hour while I do this, and then inevitably you get a phone call 10 minutes into it.

010

I suppose compared to medication or whatever, I suppose it’s investing the time, finding the time. Taking a pill is very quick, isn’t it.

002

##### IFQ Data

This was also a very common theme in the feedback questionnaire responses, with participants explaining how and why they found it difficult to find time to use the intervention (both inside and outside the workplace):

Finding the best time to do the intervention without interruption from my preschooler Vs being too tired to pay it proper attention to get the most out of it.

IFQ001

Surprisingly difficult to find 20 mins in busy clinical day or family time daily to do game.

IFQ003

My concern is that staff wouldn’t find the time during the working day to work on the interventions and with shift patterns may struggle to include it in their free time.

IFQ036

Three of the feedback questionnaire respondents also highlighted how they found it difficult to remember to record and use the intervention, in part because of the lack of time:

Remembering to do the intervention regularly and record the relevant information. During the day, I don’t have time to work on the intervention so I've had to work time into my evening to complete the tasks.

IFQ036

### Improvements and Optimizations

Participants were asked about potential improvements or optimizations to the intervention as well as any challenges experienced while using it. The responses were grouped into the following themes, which were found to be relatively frequent and consistent.

#### Difficult to Focus on Mental Rotation

##### Overview

Participants emphasized the importance of recognizing that the mental rotation aspect was the focus rather than the Tetris score:

I had to concentrate quite a bit because when I first started using it I just was having fun playing the game [laughs].

006

I think we should highlight that when you do the intervention you shouldn’t really- You should focus more on the arrangement rather than aiming for a score or you should stay away from how you usually play Tetris and focus on the future blocks and the arrangement.

014

Despite recognizing this, participants often found it difficult to concentrate or focus on mental rotation:

I was thinking about myself, my thoughts were just running away, and I wasn’t really concentrating on the Tetris game.

001

I remember thinking first few times it [speed] increased I started to panic a bit because- And then you do stop focusing on the mental rotation because you just think, oh they’re all coming so quickly.

005

I think it was the pre-empting the rotation that’s coming, to rotate it in your head before it actually comes onto the screen, that’s the hard bit.

012

##### IFQ Data

This difficulty in concentrating was echoed by participants in the feedback questionnaires:

I think 15 mins is enough—my concentration starts to flag after this.

IFQ005

I found I was bored quickly.

IFQ038

#### How to Increase Focus on Mental Rotation

##### Overview

Participants provided suggestions on how to improve the intervention for future participants. One of the main suggestions was a pop-up message during gameplay to remind participants to focus on mental rotation, as participants had previously mentioned that their focus drifted from mental rotation. Participants also mentioned that the speed at which the blocks fell made it difficult to focus on mental rotation, and they suggested that capping the speed may be helpful:

I don’t know if during the Tetris game some kind of pop up could be coming up, like a reminder. Yes? A kind of- I don’t know what you could say on the reminder, but something like- I don’t know, remember to focus on the next pieces.

001

And maybe cap the speed. When it gets ridiculously fast I think you’re very aware that you’re not doing mental rotations very well.

002

##### IFQ Data

Another participant mentioned capping the speed as an improvement suggestion in the feedback questionnaire:

Cap the speed of the blocks to enable proper rotation planning.

IFQ001

#### Researcher Support Is Important

##### Overview

Researchers provided participants with support during the initial session, in which they showed the participants how to identify intrusive memory images and how to use the intervention with a focus on mental rotation. In addition, the researchers were available to provide guidance and clarity throughout the participants’ time in the study. Participants found the initial session helpful, yet they also valued having the option for ongoing support to ensure they were using the intervention correctly and to receive guidance on making adjustments if necessary.

I think from my point of view I just find it helpful to have someone explain it all to me. I mean you could just watch the videos and read the instructions online and some people will probably be fine with that. But I think I needed it, I needed someone to actually go through it all with me and to make sure I knew how to play the game and stuff like that.

006

I like the fact that I got contacted midway through because somebody had noticed that I’ve got some- One of my intrusive memories that I actually ended up dividing into two after I’d spoken to the person midway through, because it was obvious that that one was still bothering me more.

005

Yes, I think so. I think I had a couple of teething issues, but one of the ladies phoned me up, went through it, face-to-face, got me to play it whilst on the phone with me, so it was fine.

004

##### IFQ Data

The importance of the initial session with a researcher was also highlighted by a participant in the feedback questionnaire:

Needs 1-1 at beginning when 1st doing intervention so person using it knows exactly what to do.

IFQ045

#### How to Access the Intervention Independently

All participants who received the optimized intervention provided suggestions on how to make it more easily accessible independently. For example, they proposed incorporating video demonstrations showing how to autonomously identify their intrusive memory images and playing the intervention while focusing on mental rotation:

Yes, so see someone playing. And what obviously, because you cannot read their mind, but if you put like a bubble say what is doing in their mind. They are turning, they are not focusing on this, they are doing this, this.

015

Maybe- I guess, maybe giving some examples of how to break those things [intrusive memory images] down, if that makes sense. And the different ones that are just like an image, or something that’s like a little video that plays, that sort of explanation and how to write that, maybe just some sort of video of how to break that down into something.

016

#### How to Aid Incorporation of the Intervention Into Participant Lifestyle: IFQ Data

In the feedback questionnaires, a few participants explained how shorter Tetris sessions could help them incorporate the intervention into their daily lives more easily:

Perhaps if the timing the intervention had to be carried out for was shorter - I'm not sure if it would still be effective but feel 10 minutes twice a day rather than one block of 20 minutes would be easier to fit in.

IFQ009

Is the 20 minutes a specific time or could it be reduced? Sometimes difficult in a busy working day to get full 20 minutes to spend on it. What effect with say 10 minutes? Or 5...?

IFQ021

I was sometimes deterred from starting the intervention knowing that it would take up 20mins of time. It may seem more accessible if it only required 10 mins for example. It would be possible to do whilst on break at work etc.

IFQ023

#### Other Intervention Improvement Suggestions

##### Overview

In addition, participants expressed a desire for the intervention to be available as an app rather than solely on the web-based platform:

I think it would have been really good if you could have had an app. And then every time you have a memory, you just tap the app, or something, and then it- Yes, and otherwise you have to, like- By the time you get home, and log it, you’re like, how many did I have?

004

I think it will help if it’s an App that you can download, like Calm or what’s the other one- Then you can just go and open and do for 15, 20 minutes.

014

There were mixed views on the use of graphs to display intrusive memory changes, with some participants not liking graphs, whereas others appreciated the ability to track progress in this way:

I’m not a great fan of graphs to be honest.

013

And not only to see progress but to see- I guess because I could match as well, like they definitely got worse when I was on nights and I could see that, with my shift pattern and also stuff I was doing at work, just certain things that were going on and then I’d be like, I can see how that happened and where that connection is. So, it kind of makes sense to me.

016

In general, participants appreciated receiving brief daily reminders to log their intrusive memories and engage with the intervention, as long as the reminders were not excessive and did not prompt participants who had already completed the task. However, as mentioned in a previous subtheme, some participants did find the reminders distressing as they brought the memories of traumatic incidents to mind:

A quick reminder with may be just a shortcut to the logon is fine for me.

013

[At] least I had that reminder. And I think if I forgot one day, let’s say today, tomorrow in the morning I could go back and do it...Especially when you have so many things on your mind.

015

##### IFQ Data

In addition to requesting that the intervention be available in an app format, a couple of participants in the feedback questionnaire suggested that the intervention use only 1 platform instead of 2 (1 for the intervention and 1 for logging outcome measures):

I found it difficult to navigate the website, perhaps an app would have been better.

IFQ022

It might be easier to make it all on one platform. Rather than 2 different places.

IFQ028

Although some participants mentioned how they appreciated the text message reminders, 1 participant explained in the feedback questionnaires how reminder messages could be confusing:

I would suggest keeping up the automatic reminders to record and use the intervention.

IFQ025

Infrequent but relevant text message feedback was useful and not overly intrusive.

IFQ032

I get multiple reminders about completing tasks which sometimes confuses me whether I have completed it or not.

IFQ053

## Discussion

### Principal Findings

This qualitative study explored barriers and facilitators to the adoption of a brief digital imagery-competing task intervention (1 guided intervention session of 1 hour, followed by self-guided use of approximately 20 min/session) to reduce intrusive memories of traumatic events from working in an NHS ICU during the COVID-19 pandemic. Overall, on the acceptability questionnaire, the health care staff found the intervention easy to use, helpful, and highly acceptable. They were highly willing to use the intervention and were confident in recommending it to colleagues and their health care organizations for staff exposed to repeated trauma. In the qualitative data collection, participants described many additional positives of the intervention, such as it being easy to use, enjoyable, and encouraging, as participants were able to track intrusive memories and notice reductions in frequency. They could modify the use of the intervention based on the intrusiveness and frequency of the traumatic memories. Compared with sessions of psychological treatment, it was considered less time consuming, more flexible when it could be used, did not require discussing unpleasant memories, and required less effort. Compared with medication, it was more specific in its effect on intrusive memories of traumatic events and did not have adverse effects on weight, sleep, or alertness. It was seen as complementary to psychological and medication treatments in those who needed them.

Although it has its advantages, participants described how the intervention may not entirely replace the need for psychological therapy to talk about the nature of intrusive memories in those who wish to or the need for medication in some instances. A key finding was that some participants preferred not to access the intervention through their workplace or for colleagues to know that they were using the intervention owing to mental health stigma, a factor that is known to affect mental health help seeking among health care professionals [35], including after witnessing trauma in the workplace [36]. An advantage of the GAINS intervention is that although it could be provided through the workplace and introduced as a normal working practice for staff in the ICU, it could also be accessed independently outside of work.

The intervention use data showed that, on average, staff engaged with the Tetris component for the full 20 minutes per session, approximately 7 times over the 4-week period. They were able to target approximately 73% (3.64/4.88) of their intrusive memories through the intervention, that is, on average, participants were able to target 3.64 intrusive memories and had 4.88 intrusive memories listed. This emphasizes that the intervention was extensively used, indicating its significant value. When combined with qualitative findings, it appears feasible and acceptable for staff, particularly in the short term. However, there is a need to further investigate how participants use the intervention for a longer term, particularly whether it can easily fit into their daily lives.

### Comparison With Prior Work

The findings highlighted barriers that ICU staff experience when accessing support for their mental health, such as stigma, feeling weak for seeking help [37], questioning if they were bad enough to warrant such help, and not wanting colleagues to know that they were struggling. This is consistent with previous findings investigating mental health in health care professionals [35,36] and a culture of not showing weakness in health care work settings [29]. Participants suggested that these barriers could be partially overcome by normalizing intrusive memories after trauma through testimonials from other ICU staff who participated in the GAINS study. In addition, as discussed in the existing literature [35,38], the anonymity of the intervention was important, as it was completely separated from the health care professionals’ workplace or colleagues. This suggests that staff should have the option to access the intervention through routes other than only the workplace. However, participants in the IFQ suggested that health organizations would benefit from the intervention being endorsed by senior staff members. This endorsement could occur during induction and appraisal meetings involving junior colleagues, especially in environments where staff are repeatedly exposed to trauma. If staff did find it acceptable for the intervention to be used in their work environment, it could even be incorporated into staff induction and colleagues could support one another through a “buddy system.”

In a previous meta-synthesis of digital health interventions for mental health [39], one of the key barriers to the initial approach was skepticism about how helpful a remote treatment could really be. This was also the case with the GAINS intervention. The initial skepticism was compounded by it being a simple and novel gameplay intervention, with some participants expecting the intervention to be at best a short-term distraction while they played the game. In fact, many participants went on to report long-lasting effects on the frequency and intrusiveness of their traumatic memories. Publicizing research evidence, discussing the mechanism of action of the intervention, and testimonials from ICU staff were suggested as counters to this possible skepticism. This is consistent with the literature, which highlights the importance of users being on board with digital health interventions’ aims and understanding their purpose [40]. The suggestion to publicize research evidence and provide testimonials from ICU staff is of particular importance, as prior findings emphasize that endorsement from health care professionals is valuable and helps digital health interventions to be trusted and viewed as worthwhile [40].

There were also some factors that negatively affected participants’ experiences. In our specific population of ICU staff, it was difficult for some to find time at work to use the intervention because of their busy schedules, shift patterns, and other responsibilities, particularly while working long shifts during the pandemic. Most staff were married and had caring responsibilities, so they were trying to fit the intervention in their busy schedules, for example, on public transport commutes to work. Some struggled because they were tired, often distracted, or had technological or interoperability issues across the devices and connection points. Nevertheless, reminders to use the intervention were appreciated by the participants, as suggested by Patel et al [39]. Many also preferred not to use the intervention at work and did not have access to a personal device. Therefore, it is evident that although the intervention is currently available for use on other devices (such as mobile phones), it needs to be easier to use on these devices and not be too time consuming. Borghouts et al [38] also found that engagement improves if users are able to integrate the intervention into their daily life.

Similar to prior findings [41] that integrating a human component into treatment helps retain engagement and reduce dropout, participants reported that researcher support, both before using the intervention (eg, the initial guided session) and throughout intervention use (eg, booster session), was found to be extremely helpful and important. They valued the continuous support provided to ensure they correctly used the intervention and received guidance on making adjustments when necessary. However, providing this level of support can be difficult when scaling up an intervention [41]. Participants provided suggestions on how the intervention could be more easily accessed independently, which would require fewer therapist and researcher resources and enable the intervention to spread more widely and reach a greater number of ICU staff, for example, by providing video demonstrations of someone identifying the intrusive memory images independently and playing the intervention while focusing on mental rotation. Participants’ suggestions around helping to retain a focus on mental rotation while playing Tetris are helpful to identify, as this may be one of the core aspects to the working of the intervention. They also discussed changing aspects of the game itself and whether more frequent but shorter use of the game might be effective and more feasible for staff to continue using it for a longer term.

### Limitations

Limitations of the study include the method of recruitment and sample representativeness and the short duration of use of a novel intervention. The trial was a first trial that is being followed by further trial work to test the robustness of the findings of the first trial. Recruitment of participants through advertising in the ICS, a professional organization, may have recruited participants who would be the most receptive and enthusiastic for such interventions. The sample was geographically drawn from many parts of the United Kingdom and was representative of the NHS at large but appears to have been overrepresentative of ethnic minority staff. There were 44% (7/16) staff from ethnic minority backgrounds in our interview sample compared with 20.7% (248,400/1,200,000) in the NHS workforce [42]. However, selective sampling was used for interview recruitment to capture a broad range of experiences of using the intervention from as diverse a sample as possible, rather than to match the sample to demographic characteristics of the NHS population. Any novel intervention, both in format and purpose, may have a large halo effect in relation to enthusiasm to take it up and use that may not be sustained over time. There is a need to recruit larger representative samples that use this intervention. The aim of this intervention is for most individuals to only need to use it a few times (once for each distinct intrusive memory of trauma). It is designed to be brief each time it is used, and requiring only a few sessions, rather than for prolonged use like a mindfulness app. However, for people with a very large number of intrusive memories and repeated ongoing traumatic events, it would be useful to consider use over a number of months to obtain more robust data on its likely uptake, use, and acceptability, which could be generalized to the staff experiencing repeated trauma in routine health care settings. Furthermore, we were unable to interview any ICU staff who were not able or chose not to participate in the study. Therefore, it is difficult for us to know how typical our sample and findings are of the wider ICU staff population. We also could not obtain important information about any barriers to participating in these individuals and how these barriers could be overcome, as it is likely that our participants were more open to mental health support in general. Furthermore, participants who took part in the trial but did not consent to participating in an interview may have had a different (more negative) experience of the intervention, and we could not obtain information about their experiences.

Nonetheless, our participants described potential barriers to wider participation, and it appears that the intervention was able to overcome some of these to an extent, such as the anonymity of the intervention helping to reduce the impact of stigma. In addition, we used selective sampling to ensure that our sample was as diverse as possible on factors such as profession, background, ethnicity, age, NHS Trust, and baseline intrusive memory frequencies. Therefore, our sample aimed to be as inclusive as possible of our target population. Saturation of themes was reached through the interview and analysis process, suggesting that further barriers would be unlikely to be present if we had recruited more participants.

We attempted to triangulate our data by comparing feedback from the sample that completed the acceptability questionnaire (IFQ) with our qualitative interview data. Certain topics did not lend themselves to completion on the feedback questionnaire, such as discussion of stigma. However, this was often reported in the qualitative interviews with ICU staff, and the themes resonate with previous literature. The feedback questionnaire, which had a very high rate of completion, confirmed most other barriers and facilitators, identifying a new subtheme around highlighting a number of ways staff improved cognitive and emotional coping with trauma through the intervention. A limitation of our analysis is the inability to delve deeper into certain findings. For example, we could not explore how the intervention might induce relaxation nor whether the distraction and improved focus persisted beyond the gameplay or were solely experienced during the game sessions. Bringing awareness to the intrusive memories could be both a positive and a negative experience, as it might help identify a source of stress; however, some people cope with intrusive memories by suppressing them, whereas others believe it adds to distress. The feedback questionnaire provided many additional suggestions to improve the uptake, feasibility, and acceptability of the intervention that the research team and developers of the intervention could explore and consider. A key strength of the qualitative interviews was the chance to iterate findings to adapt the intervention accordingly while it was still being used in the RCT. We also interviewed some participants who received the optimized intervention to gain feedback about their experience with the optimizations so that we could further improve the intervention.

Data gathered from the use of a survey in addition to interviews demonstrate that even when interviews are repeatedly producing the same subthemes and themes, and despite maximum variance sampling on the basis of characteristics available to us, there might still be important themes that might be missed because of the limits on maximum variance sampling imposed by data protection and trial procedures. We could only be made aware of a limited amount of information without fully consenting individuals for the interview. However, the qualitative interview method delves deeper into extracting information that participants might not readily provide in a feedback survey. In addition, it is an iterative process that builds upon multiple interviews. Therefore, if a theme or subtheme is not supported in the survey feedback, it does not mean that it is unimportant or even uncommon, simply not as immediately obvious to the participants.

### Future Research

Other potential issues for us to consider in the next phase are related to the practicalities of the intervention, as participants mentioned that the intervention could be difficult to fit into their extremely busy working lives during the pandemic. Health care demands have remained high; therefore, this ability to fit in may be a continued factor to consider. There was also the issue of lack of privacy when accessing the intervention at work and not having access to a personal device in this setting. As highlighted in a previous literature review [43], most intervention frameworks recognize the importance of understanding how well an intervention fits with existing organizational routines to predict its adoption and implementation on a larger scale. As the intervention can be accessed by ICU staff either at work or outside of work, we must also understand how well the intervention fits into their personal lives. Although this aspect was discussed in our findings, it is crucial to further explore the feasibility of long-term intervention use, especially considering that ICU staff regularly encounter work-related trauma. For the intervention to be beneficial, it must integrate as seamlessly as possible into their lives. An advantage of this intervention is that it can be used at any convenient time (eg, at home or on a commute). A further key issue going forward within work, and especially outside work, is data protection of sensitive information that may require training or other safeguards, for example, if staff members are overlooked while examining graphical outputs (eg, a line graph) of the frequency of traumatic memories. We may need to provide alternative ways of presenting the data to ensure that they are more widely accessible, such as through color chart indicators rather than numerical graphs.

### Conclusions

Overall, the data suggest that the intervention to reduce intrusive memories after trauma is highly acceptable to ICU staff and has some unique value compared with other current approaches to staff mental well-being. Through additional refinement and gathering evidence regarding outcomes and implementation, this intervention could potentially present a much-needed approach to address the widespread issue of repeated exposure to trauma, which manifesting as intrusive memories significantly impacts on the mental health and emotional well-being of health care staff.

## Appendix

Multimedia Appendix 1 Participant information sheet.

Multimedia Appendix 2 Intervention feedback questionnaire.

Multimedia Appendix 3 Interview topic guide.

## Abbreviations

GAINS: Brief Gameplay Intervention For National Health Service Intensive Care Unit Staff Affected By COVID-19 Trauma
ICS: Intensive Care Society
ICU: intensive care unit
IFQ: Intervention Feedback Questionnaire
NHS: National Health Service
PTSD: posttraumatic stress disorder
RCT: randomized controlled trial
