# Supplementary material for: Using a Novel Gameplay Intervention to Target Intrusive Memories After Work-Related Trauma: Iterative Qualitative Analysis of Intensive Care Unit Staff Experiences
Source: JMIR Form Res. 2024 Feb 29;8:e47458. doi: 10.2196/47458 (PMC10940990; doi:10.2196/47458)
Supplement: Multimedia Appendix 1 [file formative_v8i1e47458_app1.docx]

## **Participant Information Sheet**

## **Study short title: A brief GAmeplay Intervention for NHS ICU Staff affected by COVID-19 trauma (GAINS Study)**

**Long title: A randomised optimisation study of a brief digital imagery-competing task intervention to support NHS ICU staff experiencing intrusive memories of traumatic events from working in the COVID-19 pandemic.**

## **Study Number: P1V-GAINS-IN01**

**Invitation to take part in the study**

You are being invited to take part in a clinical research study. Please take a few minutes to read what it is about before you decide to participate.

**What is the purpose of the study?**

As a member of NHS Intensive Care Unit (ICU) staff you have probably been exposed to frequent significant, stressful or traumatic events during the COVID-19 pandemic. Whilst these events are common at work, the increased number compared to usual is a challenge. Many staff have subsequently described intrusive unwanted memories of these events with images that pop into their mind’s eye suddenly, such as seeing a patient’s face or an item of equipment. These intrusive memories can be fleeting but distracting so that they affect one’s concentration and the ability to do everyday tasks.

A brief online intervention, which includes playing a 20-minute computer game (Tetris), has previously been found to reduce the number of intrusive memories for people who have had a traumatic road traffic accident, emergency caesarean section, and other traumatic events. Our study will determine whether this approach can reduce the number and the impact of such intrusive memories for ICU staff. The online intervention takes about 40 minutes in a first session guided by a researcher (by video call), and after that can be done independently in your own time. There is no need to meet in person.

**Who can take part?**

To take part you will have experienced at least one traumatic event related to your work in a NHS Intensive Care Unit (or equivalent) during the COVID-19 pandemic and be currently experiencing intrusive memories of the event(s) (at least 3 per week). You will be 18 years or over and be able to read, write and speak in English.

**Do I have to take part?**

No – it is entirely up to you to decide whether or not to take part. If you do decide to take part, please keep this information sheet (e.g. save it on your phone/computer) and a researcher will contact you to arrange a video/phone call to obtain your consent using an electronic signature. You can still decide to stop participating at any time if you change your mind, and without giving a reason. A decision to withdraw at any time, or a decision not to take part, will not affect your employment or future medical care.

**What will happen to me if I take part?**

You can complete the entire study remotely on a computer, smartphone or tablet.

The whole study lasts up to 17 weeks.

There will be at least two virtual visits with a researcher (e.g. via video call using Microsoft Teams) but no inperson meetings.

You will be asked to complete online questionnaires on three occasions.

**We have broken down the study so that you can understand what it involves and exactly how long it will take:**

**1. Your eligibility: We will check if you are eligible to start the study and ask for your consent (5 minutes online and 30 minutes by phone/video call)**

- After you have read this information sheet on the study website, you will be asked to complete a brief online questionnaire anonymously to indicate if you are eligible to take part in the study. We will ask you to give consent to fill in this questionnaire using a checkbox.
- If you are eligible to take part in the study, you will be asked to provide your name, telephone number and/or email address, depending on how you would like to be contacted. A study researcher will then contact you to arrange a suitable time to go through this information sheet with you over the phone or by video call. You can ask any questions you may have to be sure that you understand the content and procedures.
- If you agree to take part, you and the study researcher will sign the consent form located at the end of this document using a simple electronic signature via email. The study researcher will go through the study inclusion and exclusion criteria with you, ask you for additional contact details and will send you an electronic copy of the consent form to keep.
- If you are not eligible to take part in the study, we will let you know where you might find support or advice if you need it.

**2. Confirming eligibility: we will ask you to briefly** **record how many intrusive memories you have each day for a week (approx. 1 minute per day plus 3 minutes at the end of the week, online)**

- Over seven days, we will send you a reminder each day by text or email (as you prefer) with a link to fill in a brief online questionnaire of how many intrusive memories you have had that day using a simple app. This will take up to 1 minute per day.
- At the end of the seven days, you will be asked to complete a questionnaire which asks about the impact of your intrusive memories over the past week. This will take about 3 minutes to complete.
- After the seven days, we will let you know if you are eligible to continue with the study. If you are not eligible to continue, we will delete your contact details and send you details of where you might find appropriate support or advice if you need it.

**3. Establishing a baseline and entering the study: if you are eligible, we will ask you to fill in some questionnaires (20-30 minutes online)**

- We will send you a link to fill in some online questionnaires. These questionnaires ask about your demographic details and health, your experience of the stressful or traumatic event(s) you encountered at work, difficulties you may be experiencing, and how you feel about your work. These will take about 20 to 30 minutes to complete.
- You will then be randomly allocated to decide when the intervention will start. The study researcher will contact you to inform you of the next steps.

1. **We may ask you to record again how many intrusive memories you have each day for one week before you start the intervention** **(approx. 1 minute per day plus 3 minutes at the end of the week, online)**.

1. **Using the online intervention for the first time guided by a researcher (40 minutes by video call)**

- At some point within 1-5 weeks after screening you will be contacted by the study researcher to arrange a suitable time to go through the online intervention for the first time by video call. This will take about 40 minutes. During the intervention you will be asked to:
  1. Briefly list your different intrusive memories (i.e. the mental images you have)
  2. Briefly bring to mind one of these images
  3. Receive instructions on how to play the computer game Tetris
  4. Play Tetris for 20 minutes
- You will be asked to briefly rate how you are feeling once before bringing to mind the image, once after bringing to mind the image and once after playing Tetris. The session will be audio recorded for training and assessment purposes.

**6. Accessing the online intervention for 4 weeks in your own time (approx. 25 minutes each time as often as you like) and recording how many intrusive memories you have each day for 4 weeks (1 minute per day plus 3 minutes at the end of the 4 weeks, online)**

- After the first time, you can do the intervention as many times as you like during the next 4 weeks to target different intrusive memories. This will take about 25 minutes each time, and you can do it on your own or with help from a researcher.
- During this time we will send you reminders by text or email (as you prefer) to complete a daily intrusive memory diary (to record how many intrusive memories you have each day).
- At the end of the last week of accessing the intervention we will email you a link to complete a brief 3minute questionnaire, which asks about the impact of your intrusive memories over the past week.

**7. We will ask you to fill in questionnaires twice more (15-20 minutes each time, online)**

• Approximately 4 weeks and 8 weeks after completing the first set of questionnaires, we will email you a link to fill in some online questionnaires again, which are similar to the first set of questionnaires. These will take about 15 to 20 minutes to complete.

**8. You will have the option of completing a feedback interview (approximately 30 minutes by phone/video call)**

• After your access to the intervention, you will have the option of meeting a researcher (e.g. via phone or video call) to complete a follow up interview. The researcher will ask you a number of questions about your experience of using the intervention including suggestions for improvement and potential barriers and challenges that you may have encountered. The interview will be audio recorded and will take about 30 minutes to complete.

**9**. **You will have the option of continuing to access the intervention for a full year after the completion of the trial if you would like to.**

**Expenses and payments**

If you decide to take part in the study, you will be offered a £20 voucher after completing the last set of online questionnaires and a £10 voucher for taking part in the optional follow up interview (after approximately 3 months), in appreciation of your help with the study.

**What will I have to do?**

As a participant in this study, we ask that you:

- Be willing and able to complete the study procedures.
- Be willing and able to be contacted by the research team during the study period.
- Have internet access.

**Which parts of the study are different to the clinical care I would normally receive?**

The online intervention and the questionnaires you complete are not normally part of clinical care.

**What are the possible side effects, disadvantages and risks of taking part?**

There are no major risks associated with taking part in the study. Some people may find briefly listing and bringing to mind their intrusive memories distressing. However, this is very brief, and we will not ask you to think about the traumatic events in any detail. This procedure has been tested in several previous studies with no adverse consequences. A clinical psychologist will go through the intervention with you the first time you use it and will be available to help with the intervention throughout the study. Most people find the intervention easy, helpful and not very distressing. If we learn important new information about possible risks or other information that might affect your willingness or ability to continue in the study, then we will tell you about it. If you take part in this study it is very important that you report possible side effects, other health changes and/or changes to medical treatments to your researcher as soon as possible.

**What are the possible benefits of taking part?**

By taking part in this study, you may have fewer intrusive memories of stressful/traumatic events you have encountered at work. Our previous research has shown that the intervention can reduce intrusive memories after different types of traumatic events. We are conducting this study to find out if the intervention also reduces intrusive memories for NHS ICU (or equivalent) staff who have experienced traumatic events in their line of work during the COVID-19 pandemic.

**What if relevant new information becomes available?**

We will tell you if any new information becomes available which may affect your decision to continue in the study. We will give you an updated information sheet and discuss this with you. If you decide to continue in the study, you will be asked to electronically consent to an updated consent form.

In some cases, we might consider it to be in your interest to withdraw you from the study. If this occurs, we will explain the reasons to you.

**What will happen if I don’t want to carry on with the study?**

You can leave the study at any time. Your participation in this research study is voluntary (your choice). If you decide to withdraw from the study, please tell us immediately using the contact details listed on the last page of this information sheet. If you do withdraw after taking informed consent, we will use any anonymous data collected up to the time of your withdrawal.

**Withdrawal from the study**

It is possible that the researchers may decide to withdraw you from the study if the principal investigator considers it necessary. Possible reasons for this are:

- Ineligibility (either arising during the study or retrospectively having been overlooked at screening)
- An adverse event that results you no longer being able to comply with study procedures.
- Significant protocol deviation
- Withdrawal of consent

**What if there is a problem?**

**Complaints**

If you have a concern about any aspect of this study, or if you need further information, you can contact the study team on +44(0)1865 522508 or gains@p1vital.com, and we will do our best to answer your questions. If you remain unhappy and wish to complain formally, please contact Dr Lalitha Iyadurai on +44(0)1865 522508 (Chief investigator).

**Harm**

Any participation in a clinical study involves a risk, however small it is. Even if there is no fault, the sponsor accepts responsibility for damage caused to the participant (or in the event of death, his/her dependants) and directly or indirectly linked to his/her participation in the study. The sponsor has taken out insurance for this responsibility.

**Will my taking part in this study be kept confidential?**

Yes, your taking part in the study will be kept confidential. If you have any high levels of distress or (e.g. thoughts about suicide or harm to others) during the study, we may encourage you to discuss this with your GP.

We are not obliged to inform your employer or manager of any of these details. If at any point during the study you reveal information that suggests professional malpractice, we will encourage you to report this to your Hospital’s Freedom To Speak Up (FTSU) Guardian and/or to follow your Trust Whistleblowing Policy.

The information you provide during the study is the research data. Any research data from which you can be identified (e.g. name, contact details, audio-recording) is known as personal data. You will be assigned with a unique identification code, and research data will be stored under this code. Personal data including your contact details (with the exception of consent records) will be stored separately from other research data in password-protected files and will be kept for a maximum of 6 months after the end of the study, after which time it will be destroyed (files will be deleted). Audio-recordings will be stored under your identification code in password-protected files and will be destroyed once they have been transcribed (i.e. written out). Transcripts of audio-recordings will be edited to remove identifying information and stored with other research data. All data will be kept either in locked filing cabinets or on sponsor password-protected network. We are obliged to keep research data (including consent records and questionnaire/intervention data) for a period of at least 3 years after final publication/public release, and de-identified data may be archived in an online repository.

If you take part in this study, you will be asked to set up a user account (with a username and password) to complete the study intervention and questionnaires online. During the study, questionnaire and intervention task data will be collected from you and stored electronically on the P1vital® products ePRO and i-spero^®^ systems. P1vital Products is fully compliant with the General Data Protection Regulation and Data Protection Act 2018 and have appropriate data security policies and procedures in place. Data (including personal identifiable data) will be stored securely on these servers and deleted at the end of the study. Your personal identifiable data will be deleted automatically from ePRO and i-spero once you have completed the study.

If you decide to stop taking part in the study part way through, we will not collect any further information from you, but will keep any information we have already collected and use it in this research. We may choose to quote something you have said word for word in a publication arising from this study, or in our recruitment materials. If we do this, we will first make sure that any identifying information is removed or disguised. We may also share data with Uppsala University in Sweden, University of Nottingham in the UK or other interested researchers but only through a pseudonymised database after removing all information that identifies individuals.

By signing this form, you understand that the study team will be collecting and using personal data about you for the study. You have the right to access and receive a copy of your personal data, and other supplementary information upon request. You are entitled to ask the study researcher what data are being collected about you and their use in connection with the study.

**What will happen to the results of the research study?**

The results of the research will may be written up for publication in scientific journals and may be presented at scientific conferences and public events. Any research publication would not identify you individually. At the end of the study, we will send you details of where you can access the published results once they become available.

Any anonymised data collected from your intervention use after you have completed the trial, will only be used to optimise the intervention and will not be considered research data.

**Participating in future research**

If you agree to be contacted regarding future research, we will hold your contact details in a password-protected database until they are no longer required. Your contact details will be kept separate from the study data and will be held on a password-protected sponsor computer/network. Contact about future research will come from our research team in the first instance. Agreeing to be contacted does not oblige you to take part in future research, and you can be removed from this register at any time you wish.

**Who is organising and funding the research?**

P1vital^®^ Products Ltd., Manor House, Howbery Park, Wallingford, Oxfordshire, OX10 8BA, UK, is funding this research and is called the ‘Sponsor’. P1vital^®^ Products Ltd. is supported by additional funding from Wellcome Trust discretionary project grant award (223016/Z/21/Z) in mental health. P1vital^®^ Products Ltd is the controller of the data. Administrative and organisational support for the study will be provided by P1vital^®^ Products Ltd., a company specialising in digital mental health solutions for the pharmaceutical and healthcare sectors.

**Who has reviewed the study?**

The study has been reviewed and approved by the Wales Research Ethics Committee 6.

It is the task of the Ethics Committees to protect people who take part in a clinical trial. They make sure that your rights as a patient and as a participant in a clinical study are respected, that based on current knowledge, the balance between risks and benefits remains favourable to the participants, that the study is scientifically relevant and ethical. You should not under any circumstances take the favourable opinion of the Ethics Committee as an incentive to take part in this study.

**Further information and contact details**

If you would like general information about research, this can be found on many websites including [www.crncc.nihr.ac.uk/ppi/ppi](http://www.crncc.nihr.ac.uk/ppi/ppi) involve.

If you would like more information about this study and whether you should participate, please contact one of our study team on the number given below. You could also ask family, friends and your GP about whether to take part. Throughout the study you can contact us to answer questions.

You will be given a copy of this information sheet and a signed consent form to keep.

**Contact details for Dr Lalitha Iyadurai and her study team:**

**Email: gains@p1vital.com**

**Tel: +44(0)1865 522508**

***Thank you for taking the time to read this.***
