# Supplementary material for: Using a Novel Gameplay Intervention to Target Intrusive Memories After Work-Related Trauma: Iterative Qualitative Analysis of Intensive Care Unit Staff Experiences
Source: JMIR Form Res. 2024 Feb 29;8:e47458. doi: 10.2196/47458 (PMC10940990; doi:10.2196/47458)
Supplement: Multimedia Appendix 2 [file formative_v8i1e47458_app2.docx]

**Intervention Feedback Questionnaire**

| **IFQ0101**  How easy did you find it to use the intervention?  (0 = not at all, 10 = very) |
| --- |
| **IFQ0102**  How helpful did you find the intervention?  (0 = not at all, 10 = very) |
| **IFQ0103**  How burdensome did you find the intervention?  (0 = very, 10 = not at all) |
| **IFQ0104**  How distressing did you find the intervention? (0 = very, 10 = not at all) |
| **IFQ0105**  Overall, how acceptable did you find the intervention?  (0 = not at all, 10 = very) |
| **IFQ0108**  If you were having intrusive memories in the future, how willing would you be to use the intervention if it was offered to you as something that would help?  (0 = not at all, 10 = very) |
| **IFQ0109**  If a colleague or friend was having intrusive memories, how confident would you be in recommending the intervention to them?  (0 = not at all, 10 = very) |
| **IFQ0110**  How much do you feel that this intervention could be used within NHS Trusts/healthcare organisations to support staff who have experienced work-related traumatic events?  (0 = not at all, 10 = very) |
| IFQ0111  How could we **improve** this intervention as something that could be offered to healthcare staff to help reduce intrusive memories after a work-related traumatic event?  (Free-text response) |
| IFQ0112  Do you have any other suggestions or comments about the intervention?  (Free-text response) |
