# Supplementary material for: Using a Novel Gameplay Intervention to Target Intrusive Memories After Work-Related Trauma: Iterative Qualitative Analysis of Intensive Care Unit Staff Experiences
Source: JMIR Form Res. 2024 Feb 29;8:e47458. doi: 10.2196/47458 (PMC10940990; doi:10.2196/47458)
Supplement: Multimedia Appendix 3 [file formative_v8i1e47458_app3.docx]

**Topic Guide for the GAINS Qualitative Optimisation and Feasibility Interview**

**Overall views**

- What are your views on this digital intervention, how did you find it?

**Ease-of-use**

- How easy was it to use the digital intervention? What was easy or helpful about it?

- Was anything about the digital intervention difficult or challenging? If so, how could we improve this?

- How easy or difficult was it to incorporate this digital intervention into your day?

- How familiar are you with using apps or games, and have you played Tetris before?

- How did it feel to use a digital intervention that included a computer game?

- How does this digital intervention compare to other types of support that are available, such as talking therapies or medication? What do you feel are some of the specific advantages? What do you feel are some of the specific disadvantages?

- Do you think you would have felt comfortable having this conversation about intrusive memories prior to using the digital intervention?

**Type of use and impact**

- How did you use the intervention? How often did you use it? At what times and under what circumstances did you use the intervention? When and where did you use it? If not often, why was this?

- Have you experienced a change in the number of intrusive memories you are experiencing? If so, how has this affected how you function day-to-day and at work?

- Have there been any changes as a result of the digital intervention? E.g. in what you do or how you work

**Support**

- How clear were the instructions provided on how to use the intervention? How could they be improved?

- How helpful was the first training session with a researcher when you started the digital intervention? How could it be improved?

- How necessary was this first session? How would you feel about accessing the digital intervention on your own without researcher support?

- How would you prefer to use this digital intervention (entirely independently, with assistance or with optional assistance)?

- If you were to access it on your own, what else would the intervention require? [Optimised Interviews only]

- How would you feel about identifying images on your own? What would help you to identify images independently or play Tetris in the right way? [Optimised Interviews only]

- What else would be helpful if you were using the intervention independently (without researcher support)? [Optimised Interviews only]

- How would you feel about showing a friend or colleague to use this digital intervention?

**Study processes**

- Which NHS Trust do you work in?

- How did you hear about the study? What information about the study is important to help you decide whether you would like to find out more? What information would you like to see first and how (Twitter, other social media, ICS newsletter, text message)? And on the website? Was there anything specific that helped you decide to take part in the study or anything that put you off taking part?

- How did you find the way in which you were contacted?

- Which contact method would you most prefer – through your employer or through peers, professional organisations (like ICS) or social media? And which do you think your colleagues would most prefer? (And why?)

- How did you find filling in a diary with your intrusive memories every day? Did you find it helpful or unhelpful?

- How helpful were the intrusive memory graphs? How did you use them? [Optimised Interviews only]

- What types of reminders do you find useful? Did you prefer the automated system reminders or more personal reminders (e.g. the text messages)? [Optimised Interviews only]

**Future usage**

- Can you describe what intrusive memories are in your own words? If you were telling a friend how to use the intervention by themselves, how would you describe what to do?

- Are there any aspects of the digital intervention that would put you off using it again in the future?

- Are there any aspects of the digital intervention that make you more likely to use it again in the future?

- Can you foresee any challenges when providing this to other healthcare staff, such as encouraging them to access support for themselves as well as supporting other people? If so, how do you think these challenges could be addressed?

- Do you have any further comments to make on the study or the digital intervention?

- Would you recommend the study to any of your colleagues or friends If so, what are the features that you would highlight? If not, what reasons would you give them?
